# Health literacy and gout characteristics in a primary care cohort

**DOI:** 10.1093/rap/rkae034

**Published:** 2024-03-06

**Authors:** Lorraine Watson, Joanne Protheroe, Christian D Mallen, Sara Muller, Edward Roddy

**Affiliations:** School of Medicine, Keele University, Keele, UK; Midlands Partnership University NHS Foundation Trust, Staffordshire, UK; School of Medicine, Keele University, Keele, UK; School of Medicine, Keele University, Keele, UK; Haywood Academic Rheumatology Centre, Midlands Partnership University NHS Foundation Trust, Stoke-on-Trent, UK; School of Medicine, Keele University, Keele, UK; School of Medicine, Keele University, Keele, UK; Haywood Academic Rheumatology Centre, Midlands Partnership University NHS Foundation Trust, Stoke-on-Trent, UK

**Keywords:** gout, health literacy, flare, serum urate, urate-lowering therapy

## Abstract

**Objectives:**

To examine the cross-sectional association between health literacy and gout characteristics.

**Methods:**

In a primary care cohort of adults living with gout, the prevalence of poor health literacy was defined using the Single-Item Literacy Screener (SILS). Multiple logistic regression was used to obtain adjusted odds ratios (ORs) for the cross-sectional associations between health literacy and individual gout characteristics (frequency of flares, age at gout onset, history of oligo-/polyarticular flares, allopurinol use, allopurinol dose and serum urate level) with 95% CIs and adjustment for age, sex, deprivation and further education.

**Results:**

Of 551 participants [mean age 54.4 years (s.d. 11.2), 498 (90.4%) male], 163 (30.1%) reported two or more flares in the previous 12 months. Fifty-one (9.4%) had poor health literacy. Poor health literacy was associated with having two or more flares in the preceding 12 months [adjusted OR 4.10 (95% CI 2.04, 8.19)] and a history of oligo-/polyarticular flares [OR 1.93 (95% CI 1.06, 3.55)]. No associations were identified between health literacy and age at gout onset [OR 0.99 (95% CI 0.96, 1.01)], allopurinol use [OR 0.88 (95% CI 0.46, 1.65)] or dose [OR 1.00 OR (95% CI 1.00, 1.00)] or serum urate [most recent serum urate OR 1.0 (95% CI 1.00, 1.00)].

**Conclusions:**

Frequent flares and a history of oligo-/polyarticular flares were associated with poor health literacy. Since health literacy is an important determinant of health outcomes, it is important to consider health literacy when providing information and education to people with gout.

Key messages

- Poor health literacy in people with gout was associated with more frequent flares and oligo-/polyarticular flares.
- Considering health literacy is important when providing information and education to people with gout.

## Introduction

Gout is the most common inflammatory arthritis, affecting 2.5% of the UK population [1], yet is often suboptimally managed [2]. Health literacy is ‘the knowledge, motivation and competencies of accessing, understanding, appraising and applying health-related information within the healthcare, disease prevention and health promotion setting’ [3]. Poor health literacy contributes to health inequalities [4] and is associated with lower access to preventative services [5], worse outcomes from musculoskeletal pain [6], poor skills relating to taking medication and interpreting health messages [5] and detrimental effects on self-management behaviours [7].

Health literacy has been investigated in people with musculoskeletal/rheumatological conditions, including gout, in South Australia [8] and the Netherlands [9]. Qualitative studies describe poor knowledge regarding gout and its management in people with gout [10, 11]. However, there are no studies investigating the relationship between gout characteristics and health literacy. This study aimed to examine the cross-sectional associations between health literacy and gout characteristics.

## Methods

### Participants

This study used data from a 5-year prospective cohort study undertaken in primary care in the West Midlands, UK [12]. Adults ≥18 years of age registered with any of 20 participating general practices were recruited to the study if they had consulted with gout or received a prescription for allopurinol or colchicine in the preceding 2 years. Read codes, which are a coded hierarchy of clinical codes based on International Classification of Diseases, 9th Revision codes used for diagnostic coding in primary care in the UK, were used to identify gout consultations. Eligible participants were mailed a baseline questionnaire (October–November 2012) and follow-up questionnaires at 6, 12, 24, 36, 48 and 60 months. This cross-sectional analysis used health literacy data collected in the 48-month questionnaire, the only time point at which health literacy was assessed.

Written informed consent was obtained from participants who were also asked to provide consent for the practice to provide the research team with information about comorbidities, medication and investigations from their medical records. Ethical approval for this cohort study was granted by North West-Liverpool East Research and Ethics Committee (reference 12/NW/0297).

### Data collection

Health literacy was defined using the Single-Item Literacy Screener (SILS), included in the 48-month questionnaire, which asked, ‘How often do you need to have someone help you when you read instructions, pamphlets, or other written material from doctor or pharmacy?’ [13]. Response options were 1–never, 2–rarely, 3–sometimes, 4–often and 5–always, with poor health literacy defined as needing help sometimes, often or always.

Characteristics of participants were obtained from questionnaire responses, general practice data and medical records data. Age and sex were determined from general practice data at baseline. Age at gout onset, attendance at further education and ethnicity were self-reported in the baseline questionnaire. Flare frequency, history of oligo-/polyarticular flares (ever), allopurinol use and dose and global health on a 0–10 numeric rating scale were self-reported in the 48-month questionnaire. The total number of comorbidities was the sum of comorbidities (self-reported comorbidities in the 48-month questionnaire) and chronic kidney disease (CKD) stage ≥3, defined as an estimated glomerular filtration rate (eGFR) <60 ml/min/1.73 m^2^ (from the medical records). BMI was calculated from weight and height (self-reported in the 48-month questionnaire). Neighbourhood deprivation was determined from the rank of the indices of multiple deprivation (IMD) using participant postal codes (from practice data at baseline) [14]. IMD ranks were categorized into tertiles. Self-reported current job title, or last job title if not working or retired, in the 48-month questionnaire was coded to one of nine major occupational groups based on the Standard Occupational Classification 2020 [15]. Tophi, serum urate level (most recent) and eGFR (ever) were obtained from the medical records between the 2 years pre-baseline and 4 years post-baseline.

### Statistical analysis

Descriptive statistics were used to describe the characteristics of participants responding at 48 months and those with and without poor health literacy. The proportion of participants reporting poor health literacy was calculated. Logistic regression was used to obtain unadjusted odds ratios (ORs) between health literacy and gout characteristics (flare frequency, age at gout onset, history of oligo-/polyarticular flares, allopurinol use, allopurinol dose and serum urate level) with 95% CIs. Multiple logistic regression was used to obtain adjusted ORs (95% CIs) between health literacy and individual gout characteristics, adjusting initially for age and sex, and then further adjusting for deprivation tertile and attendance at further education. Multiple imputation was undertaken (see Supplementary Data S1, available at *Rheumatology Advances in Practice* online) to ascertain the influence of missing data. Statistical analysis was undertaken using Stata 14 (StataCorp, College Station, TX, USA).

## Results

### Participant characteristics

A total of 551 participants responded to the questionnaire at 48 months (response 63.5%; questionnaire responders/eligible participants at 48 months). The mean age was 54.4 years (s.d. 11.2), 498 (90.4%) were male, 534 (98%) were white/European and 136 (25.4%) reported attending higher education (Table 1). A total of 163 (30.1%) participants reported having two or more flares in the previous 12 months, with 209 (38.2%) reporting a history of oligo-/polyarticular flares (Table 2). Twenty-four (4.5%) had tophi. A total of 361 (66.7%) participants reported allopurinol use {median dose 300 mg [interquartile range (IQR) 100–300 mg]}. The mean serum urate was 399.6 µmol/l (s.d. 107.8), with a serum urate <300 µmol/l in 75 (19.7%) patients.

### Prevalence of poor health literacy

The prevalence of poor health literacy was 9.4% (*n* = 51).

**Table 1.** Characteristics of all responders and those with and without poor health literacy

Characteristics	Responders (*n* = 551)	Poor health literacy, yes (*n* = 51)	Poor health literacy, no (*n* = 492)
Age, years, mean (s.d.)	64.4 (11.2)	66.7 (11.9)	64.0 (11.0)
Male, *n* (%)	498 (90.4)	42 (82.4)	450 (91.5)
White UK/European, *n* (%)	534 (98.0)	49 (96.1)	477 (98.2)
IMD tertile, *n* (%)			
Most deprived	187 (33.9)	27 (52.9)	158 (32.1)
Middle	184 (33.4)	17 (33.3)	165 (33.5)
Least deprived	180 (32.7)	7 (13.7)	169 (34.4)
Further education attendance, *n* (%)	136 (25.4)	5 (10.0)	127 (26.6)
Occupational group, *n* (%)			
Managers, directors and senior officials	102 (19.5)	10 (21.7)	91 (19.2)
Professional occupations	107 (20.4)	5 (10.9)	102 (21.6)
Associate professional and technical occupations	63 (12.0)	2 (4.4)	61 (12.9)
Administrative and secretarial occupations	29 (5.5)	4 (8.7)	25 (5.3)
Skilled trades occupations	103 (19.7)	14 (30.4)	87 (18.4)
Caregiving, leisure and other service occupations	8 (1.5)	1 (2.2)	7 (1.5)
Sales and customer service occupations	14 (2.67)	3 (6.5)	10 (2.11)
Process, plant and machine operatives	50 (9.5)	5 (10.9)	45 (9.5)
Elementary occupations	48 (9.2)	2 (4.4)	45 (9.5)
Self-reported comorbidities, *n* (%)			
Diabetes	108 (19.6)	18 (35.3)	89 (18.1)
Cerebrovascular accident	16 (2.9)	5 (9.8)	11 (2.2)
Hypertension	331 (60.1)	40 (78.4)	288 (58.5)
Transient ischaemic attack	36 (6.5)	8 (15.7)	28 (5.7)
Hyperlipidaemia	209 (37.9)	23 (45.1)	183 (37.2)
Myocardial infarction	46 (8.4)	6 (11.8)	40 (8.1)
Renal calculi	33 (6.0)	7 (13.7)	26 (5.3)
Angina	57 (10.3)	6 (11.8)	49 (10.0)
CKD stage ≥3 (eGFR <60 ml/min/1.73 m^2^),^a^*n* (%)	241 (45.1)	34 (68.0)	201 (42.1)
Total comorbidities, median (IQR)	2 (1–3)	3 (2–4)	2 (1–3)
Global health NRS, median (IQR)	2 (0–4)	5 (3–7)	1 (0–4)
BMI, median (IQR)	28.5 (26.0–31.5)	29.5 (26.2–32.6)	28.5 (26.0–31.3)

Denominator is either the number of participants responding to the questionnaire item for each characteristic or the number with an eGFR in their medical record. Poor health literacy was defined as reporting sometimes, often or always needing help when reading printed health-related material. Occupational group based on major standard occupational codes. Total comorbidities from self-reported comorbidities and CKD stage ≥3 defined as an eGFR <60 ml/min/1.73 m^2^. Global health NRS (numerical rating scale) ranges from 0 (very well) to 10 (very poor health). BMI body mass index.

a In the medical record in the 2 years pre-baseline and 4 years post-baseline.

**Table 2.** Gout characteristics of all responders, characteristics of responders with and without poor health literacy and associations between gout characteristics and poor health literacy

Characteristics	Responders (*n* = 551)	Poor health literacy, yes (*n* = 51)	Poor health literacy, no (*n* = 492)	Crude OR (95% CI)	Adjusted OR (age, sex) (95% CI)	Adjusted OR (age, sex, deprivation^a^, further education) (95% CI)
Flares in previous 12 months, *n* (%)						
0	307 (56.8)	15 (30.6)	287 (59.2)	1	1	1
1	71 (13.1)	5 (10.2)	65 (13.4)	1.47 (0.51, 4.19)	1.59 (0.56, 4.58)	1.74 (0.59, 5.15)
≥2	163 (30.1)	29 (59.2)	133 (27.4)	**4.17 (2.16, 8.04)**	**4.57 (2.34, 8.90)**	**4.10 (2.04, 8.19)**
History of oligo-/polyarticular flares, *n* (%)						
No	338 (61.8)	24 (47.1)	306 (62.7)	1	1	1
Yes	209 (38.2)	27 (52.9)	182 (37.3)	**1.89 (1.06, 3.38)**	**2.04 (1.13, 3.68)**	**1.93 (1.06, 3.55)**
Age at gout onset, years, mean (s.d.)	51.8 (14.7)	51.5 (14.5)	51.5 (14.5)	1.00 (0.98, 1.02)	0.98 (0.96, 1.00)	0.99 (0.96, 1.01)
Self-reported allopurinol use, *n* (%)						
No	180 (33.3)	18 (36.7)	156 (32.2)	1	1	1
Yes	361 (66.7)	31 (63.7)	329 (67.8)	0.82 (0.44, 1.50)	0.86 (0.46, 1.59)	0.88 (0.46, 1.65)
Self-reported allopurinol dose, mg, median (IQR)	300 (100–300)	300 (250–300)	300 (100–300)	1.00 (1.00–1.00)	1.00 (1.00–1.00)	1.00 (1.00–1.00)
Most recent serum urate level, µmol/l, mean (s.d.)^b^^,^^c^	399.6 (107.8)	411.6 (97.8)	397.0 (109.2)	1.00 (1.00–1.00)	1.00 (1.00–1.00)	1.00 (1.00–1.00)
Serum urate <300 µmol/l^b^^,^^c^, *n* (%)						
No	265 (80.3)	30 (85.7)	231 (79.4)	1	1	1
Yes	65 (19.7)	5 (14.3)	60 (20.6)	0.64 (0.24, 1.72)	0.50 (0.18, 1.44)	0.54 (0.19, 1.60)
Serum urate <360 µmol/l^b^^,^^c^, *n* (%)						
No	208 (63.0)	23 (65.7)	181 (62.2)	1	1	1
Yes	122 (37.0)	12 (34.3)	110 (37.8)	0.85 (0.41, 1.71)	0.5 (0.18, 1.44)	0.81 (0.37, 1.77)
Serum urate recorded in medical record^b^, *n* (%)	330 (59.9)	35 (68.6)	291 (59.0)	–	–	–
Record of tophi^b^, *n* (%)	24 (4.5)	3 (6.0)	21 (4.4)	–	–	–

Statistically significant values are in bold. Denominator is the number of participants responding to the questionnaire item for each characteristic. The denominator for tophi is the number of participants consenting to a medical records review (*n* = 1079) and the denominator for serum urate is the number of participants with a serum urate in the medical records. Poor health literacy was defined as reporting sometimes, often or always needing help when reading printed health-related material.

a IMD tertile.

b In the medical record in the 2 years pre-baseline and 4 years post-baseline.

c Most recent serum urate recorded.

### Health literacy and participant characteristics

Participants with poor health literacy were more likely to be female (17.6% with poor health literacy *vs* 8.5% without poor health literacy), more deprived (52.9% *vs* 32.1%), did not attend further education (10.0% *vs* 26.6%) and did not have a professional occupation (10.9% *vs* 21.6%) compared with participants without poor health literacy (Table 1). Comorbidities were more common in people with poor health literacy. Those with poor health literacy had a higher global health score, indicating worse perceived overall health.

### Health literacy and gout characteristics

A greater proportion of participants with poor health literacy reported having two or more flares in the previous 12 months (59.2% *vs* 27.4%) and a history of oligo-/polyarticular flares (52.9% *vs* 37.3%) compared with those without poor health literacy (Table 2). Slightly more participants with poor health literacy had a serum urate level recorded in their medical record (68.6% *vs* 59.0%). Fewer participants with poor health literacy had a serum urate <300 µmol/l (14.3% *vs* 20.6%).

On unadjusted logistic regression, poor health literacy was associated with having two or more flares in the previous 12 months [OR 4.17 (95% CI 2.16, 8.04)] and a history of oligo-/polyarticular flares [OR 1.89 (95% CI 1.06, 3.38)] compared with those without poor health literacy (Table 2). These associations remained statistically significant after adjustment for age and sex [two or more flares: OR 4.57 (95% CI 2.34, 8.90); oligo-/polyarticular flares: OR 2.04 (95% CI 1.13, 3.68)] and age, sex, deprivation and further education [two or more flares: OR 4.10 (95% CI 2.04, 8.19); oligo-/polyarticular flares: OR 1.93 (95% CI 1.06, 3.55)].

No associations were identified between health literacy and age at gout onset, allopurinol use or dose or serum urate levels in either the unadjusted or adjusted analyses.

### Missing data and multiple imputation

Variables had <10% missing data, except allopurinol dose and serum urate (35.8% and 40.1% missing, respectively) (Supplementary Table S1, available at *Rheumatology Advances in Practice* online). The results did not change following multiple imputation (Supplementary Table S2, available at *Rheumatology Advances in Practice* online).

## Discussion

This study found the prevalence of poor health literacy in people with gout to be 9.4%. Participants with poor health literacy were more likely to be female, more deprived, have not attended further education, have more comorbidities and self-report poor health. More frequent flares, a history of oligo-/polyarticular flares, having a serum urate recorded in the medical records and a slightly higher serum urate were also more likely in participants with poor health literacy. In multivariate analyses, poor health literacy was associated with having two or more flares in the preceding 12 months and a history of oligo-/polyarticular flares, after adjustment for age, sex, deprivation and attending further education.

The prevalence of poor health literacy of 9.4% in our cohort is lower than that reported in people with gout in South Australia (35.8%), measured using the Newest Vital Sign [8], and with musculoskeletal pain in the UK (16%), measured using the SILS [16]. The higher prevalence observed in South Australia likely reflects this being a population-based study. Recruiting participants using a postal questionnaire, which requires them to read and understand the cover letter and survey, may have biased recruitment towards people with better health literacy than a general primary care population. Our cohort had a higher proportion of managers, directors and senior officials compared with the general UK population [17]. We previously reported that loss to follow-up in our study was higher in those living in deprived areas or who did not attend further education [18], meaning that people with poor health literacy may have dropped out prior to 48 months, underestimating its prevalence. Our study is the first to assess the association between gout characteristics and health literacy, showing that people who have more frequent and oligo-/polyarticular flares are more likely to have poor health literacy. Inadequate health literacy is associated with worse disease severity, physical function and pain in other rheumatological/musculoskeletal conditions [6, 19].

The feasibility of using the SILS to identify low health literacy was confirmed in a primary care cohort [16], however, its focus on reading and understanding written information is only one aspect of health literacy [13]. The Newest Vital Sign, which was used to investigate health literacy in people with gout in South Australia, asks participants to interpret text and perform a small numeracy task [8]. Neither measure takes account of clinician–patient verbal communication, nor the patient’s social support network, both of which may impact health literacy. The Health Literacy Questionnaire, which comprises nine domains, was used to assess people with gout in the Netherlands, who had lower scores for feeling supported by providers, actively managing health and finding and understanding health information [9].

Limitations of this study require acknowledgment. The participants were predominantly white/European and recruited from a single geographical region in England, meaning they may be unrepresentative of the general gout population, potentially limiting the generalisability of these findings. The prevalence of health literacy, and the associations observed with gout characteristics, might have been different if alternative methods to collect data or assess health literacy had been used. Gout flares were self-reported, and it is possible people with poor health literacy may interpret gout flares differently from people without. Although we demonstrated associations between poor health literacy and certain aspects of gout severity, such as flare frequency and distribution, we did not assess pain intensity, flare duration or function limitation. We did not adjust for all potential confounders between gout characteristics and health literacy, because we aimed to explore their associations and not to infer causation.

These findings are relevant to primary care, where most people with gout are managed, and to clinicians communicating with people with gout in clinical consultations. They highlight the importance of considering health literacy when providing information and education to support people with gout. Suboptimal understanding, information and education relating to gout are important barriers to gout management [10, 11, 20]. Online patient information resources on gout are commonly difficult to read [21], which is likely to be amplified for people with poor health literacy. More active utilization of online information and booklets by clinicians is needed to improve gout health literacy [22]. Health literacy was described as the most common reason for withdrawal from urate-lowering therapy (ULT) in a tertiary care cohort [23]. It is surprising that ULT use was not lower in people with poor health literacy; however, clinicians may be more inclined to offer ULT to people with poor health literacy because they have more severe gout, yet people with poor health literacy may be less likely to accept ULT, meaning that overall ULT use in those with poor health literacy does not differ.

Future studies should evaluate whether interventions to aid communication with people with lower levels of health literacy (such as ‘teach back’, encouraging patient questions and using simple language and pictures) improve clinical outcomes in patients with gout. Prospective studies could investigate whether our findings are replicated in other cohorts.

Frequent flares and a history of oligo-/polyarticular flares were associated with poor health literacy in people with gout in primary care, highlighting the importance of considering health literacy when providing information and education to people with gout.

## Supplementary Material

rkae034_Supplementary_Data

## Data Availability

Data are available upon reasonable request via open or restricted access through a strict controlled access procedure. In the first instance, data requests and enquiries should be directed to medicine.datasharing@keele.ac.uk.
