# Supplementary material for: Health literacy and gout characteristics in a primary care cohort
Source: Rheumatol Adv Pract. 2024 Mar 6;8(2):rkae034. doi: 10.1093/rap/rkae034 (PMC10997429; doi:10.1093/rap/rkae034)
Supplement: rkae034_Supplementary_Data [file rkae034_supplementary_data.docx]

**Supplementary information file**

**Health Literacy and Gout Characteristics in a Primary Care Cohort**

| Supplementary Table S1: Missing data for all responders and responders with and without poor health literacy | | | |
| --- | --- | --- | --- |
|  | Responders  n=551 | Poor health  literacy  Yes  n=51 | Poor health  literacy  No  n=492 |
| **Health literacy** | 8(1.5) | 0(0) | 0(0) |
| **Age** | 0(0) | 0(0) | 0(0) |
| **Male** | 0(0) | 0(0) | 0(0) |
| **White UK/European** | 0(0) | 0(0) | 0(0) |
| **Indices of multiple deprivation (IMD) tertile** | 0(0) | 0(0) | 0(0) |
| **Attendance at further education** | 16(2.9) | 1(2.0) | 15(3.1) |
| **Occupational group^†^** | 27(4.9) | 5(9.8) | 19(3.9) |
| **Self-reported comorbidities** | 0(0) | 0(0) | 0(0) |
| **eGFR < 60 mL/ min/1.73m^2^** ^◇^ | 16(2.9) | 1(2.0) | 15(3.1) |
| **Total comorbidities** | 0(0) | 0(0) | 0(0) |
| **Global health NRS** | 24(4.4) | 3(5.9) | 16(3.3) |
| **BMI** | 19(3.5) | 3(5.9) | 12(2.4) |
| **Flares in previous 12 months** | 10(1.8) | 2(3.9) | 7(1.4) |
| **History of oligo/polyarticular flares** | 4(0.73) | 0(0) | 4(0.8) |
| **Age of onset** | 31(5.6) | 1(2.0) | 30(6.1) |
| **Self-reported allopurinol use** | 10(1.8) | 2(3.9) | 7(1.4) |
| **Self-reported allopurinol dose** | 197(35.8) | 19(37.3) | 170(34.6) |
| **Serum urate^◇^** | 221(40.1) | 16(31.4) | 201(40.9) |
| **Record of tophi^◇^** | 16(2.9) | 1(2.0) | 15(3.1) |
| **Values are n(%)** unless otherwise stated. **Poor health literacy** defined as reporting sometimes, often or always needing help when reading printed health-related material.  **†Occupational Group** based on Major Standard Occupational Codes**. Total comorbidities** from self-reported comorbidities and chronic kidney disease (**CKD**) stage ≥3 defined as estimated glomerular filtration rate (**eGFR**)<60 mL/ min/1.73m^2^).  **Global health NRS** (numerical rating scale) ranges from 0 (very well) to 10 (very poor health). **BMI** body mass index. **^◇^**In medical record in the two years pre baseline and 4 years post baseline. | | | |

**Supplementary Data S1. Multiple imputation method**

Multiple imputation (MI) using chained equations was undertaken using Stata 14. The following variables were included in the MI model, auxiliary variables (with no missing data) were age, sex, index of multiple deprivation tertile, and imputed variables were health literacy item, attendance at further education, gout flare, history of oligo /polyarticular flares (ever), age at onset of gout, self-reported allopurinol use, self-reported allopurinol dose, serum urate. Continuous variables were imputed using predictive mean matching (pmm), dichotomous variables were imputed using logistic regression (logit) and categorical variables were imputed with ordered logistic regression (ologit). 40 imputations were undertaken for each imputed variable, as serum urate was missing at 40% in this cohort. [1] Results were pooled using mi estimate to logistic regressions, estimates were pooled using Rubin’s rule for pooling. [2]

The results of the MI can be found in supplementary table S2.

[1] White IR, Royston P, Wood AM. Multiple imputation using chained equations: Issues and guidance for practice. *Tutorial in Biostatistics* 2011; 30: 377–399.

[2] Rubin DB. *Multiple Imputation for Nonresponse in Surveys*. Wiley: New York, 1987

Supplementary Table S2: Associations between gout characteristics and poor health literacy after multiple imputation

|  | Crude OR  (95% CI) | Adjusted OR  (Age, Sex)  (95% CI) | | Adjusted OR  (Age, sex, deprivation†, further education)  (95% CI) |
| --- | --- | --- | --- | --- |
| **Flares in previous 12 months:** |  |  | |  |
| 0 gout flares | 1 | 1 | | 1 |
| 1 gout flare | 1.5(0.52,4.23) | 1.62(0.56,4.64) | | 1.64(0.56,4.85) |
| ≥2 gout flares | **4.28(2.22,8.26)** | **4.71(2.41,9.18)** | | **4.30(2.16,8.57)** |
| **History of oligo/polyarticular flares:** |  |  | |  |
| No | 1 | 1 | | 1 |
| Yes | **1.87(1.05,3.35)** | **2.04(1.12,3.67)** | | **1.84(1.1,3.35)** |
| **Age at onset** years | 1.00(0.98,1.02) | 0.97(0.96,1.10) | | 0.99(0.96,1.07) |
| **Self-reported allopurinol use:** |  |  | |  |
| No | 1 | 1 | | 1 |
| Yes | 0.83(0.45,1.5) | 0.87(0.47,1.62) | | 0.91(0.49,1.70) |
| **Self-reported allopurinol dose** mg | 1.00(1.00,1.00) | 1.00(1.00,1.00) | | 1.00(1.00,1.00) |
| **Most recent serum urate level** µmol/L^◇^* | 1.00(1.00,1.00) | 1.00(1.00,1.00) | | 1.00(1.00,1.00) |
| **Serum urate below 300** µmol/L^◇^*: |  |  | |  |
| No | 1 | 1 | | 1 |
| Yes | 0.75(0.31,1.82) | 0.60(0.24,1.52) | | 0.68(0.26,1.77) |
| **Serum urate below 360** µmol/L^◇^*: |  |  | |  |
| No | 1 | 1 | | 1 |
| Yes | 0.91(0.46,1.79) | 0.76(0.37,1.56) | | 0.88(0.42,1.82) |
| **Serum urate recorded** in medical record**^◇^** | - | - | | - |
| **Record of tophi** ^◇^ | - | - | | - |
| **Poor health literacy** defined as reporting sometimes, often or always needing help when reading printed health-related material. **Bold** indicates statistically significant values. **^◇^**In medical record in the two years pre baseline and 4 years post baseline. *most recent serum urate recorded. **OR** odds ratio. **CI** confidence intervals.† Indices of deprivation tertile. | | | | |
|  |  | |  |  |
